# Quercetin Inhibits Pulmonary Arterial Endothelial Cell Transdifferentiation Possibly by Akt and Erk1/2 Pathways

**DOI:** 10.1155/2017/6147294

**Published:** 2017-03-27

**Authors:** Shian Huang, Xiulong Zhu, Wenjun Huang, Yuan He, Lingpin Pang, Xiaozhong Lan, Xiaorong Shui, Yanfang Chen, Can Chen, Wei Lei

**Affiliations:** ^1^Cardiovascular Medicine Center, Affiliated Hospital of Guangdong Medical University, Zhanjiang 524001, China; ^2^Laboratory of Cardiovascular Diseases, Guangdong Medical University, Zhanjiang 524001, China; ^3^Guangdong Key Laboratory of Age-Related Cardiac and Cerebral Diseases, Affiliated Hospital of Guangdong Medical University, Zhanjiang 524001, China; ^4^Department of Cardiovascular Medicine, The People's Hospital of Gaozhou, Maoming 525200, China; ^5^Outpatient Operating Room, Affiliated Hospital of Guangdong Medical University, Zhanjiang 524001, China; ^6^Tibetan Collaborative Innovation Center of Agricultural and Animal Husbandry Resources, Tibet Agricultural and Animal Husbandry College, Nyingchi 860000, China; ^7^Laboratory of Vascular Surgery, Guangdong Medical University, Zhanjiang 524001, China; ^8^Department of Pharmacology & Toxicology, Boonshoft School of Medicine, Wright State University, Dayton, OH 45435, USA

## Abstract

This study aimed to investigate the effects and mechanisms of quercetin on pulmonary arterial endothelial cell (PAEC) transdifferentiation into smooth muscle-like cells. TGF-*β*1-induced PAEC transdifferentiation models were applied to evaluate the pharmacological actions of quercetin. PAEC proliferation was detected with CCK8 method and BurdU immunocytochemistry. Meanwhile, the identification and transdifferentiation of PAECs were determined by FVIII immunofluorescence staining and *α*-SMA protein expression. The related mechanism was elucidated based on the levels of Akt and Erk1/2 signal pathways. As a result, quercetin effectively inhibited the TGF-*β*1-induced proliferation and transdifferentiation of the PAECs and activation of Akt/Erk1/2 cascade in the cells. In conclusion, quercetin is demonstrated to be effective for pulmonary arterial hypertension (PAH) probably by inhibiting endothelial transdifferentiation possibly via modulating Akt and Erk1/2 expressions.

## 1. Introduction

Pulmonary arterial hypertension (PAH), a devastating disease of heart-lung syndrome, is characterized by pulmonary vasoconstriction, inflammation, and alveolar arteriole remodeling. The increase of pulmonary vascular resistance and pressure leads to right ventricular failure and even death [1]. The mortality rate of PAH remains at 40% in 5 years, and there is no effective therapeutic treatment to date [2, 3]. The stagnation of treatment is attributed to a lack of understanding of the PAH pathophysiological mechanisms and the absence of any efficacious drug against pulmonary vascular remodeling [4]. Recent studies have shown that the muscularization of nonmuscular pulmonary arterioles is a key characteristic of pulmonary vascular remodeling in PAH pathogenesis [5–9]. The new smooth muscle cells in muscularized nonmuscular pulmonary arterioles may originate from transdifferentiation and proliferation of pulmonary arterial endothelial cells (PAECs). Therefore, inhibition of PAEC transdifferentiation has the potential in the treatment of PAH [10–12].

Transforming growth factor *β*1 (TGF-*β*1), a multifunctional cytokine closely involved in cell differentiation, proliferation, and survival, can be used to induce transdifferentiation of stem cells to muscle cells and epithelial cells apoptosis by various signal pathways such as serine/threonine kinase Akt and extracellular signal-regulated kinase (ERK) [13, 14]. Recently, TGF-*β*1 has been revealed to trigger the expression of *α*-smooth muscle actin (*α*-SMA) in mature vascular endothelial cells [15–19] and to regulate the transdifferentiation progress of endothelial cells into smooth muscle-like cells [20]. Interestingly, increased synthesis and accumulation of TGF-*β*1 have been observed during progression of PAH [21, 22]. However, its role in PAEC transdifferentiation remains unknown.

Quercetin, a bioflavonoid with well-known antioxidant and anti-inflammatory activities [23], is abundant in plants including fruits, tea, and herbs. Some studies have already revealed the bioactive function of quercetin in improving heart and lung circulation. For instance, quercetin dilates blood vessels constricted by a variety of endogenous factors such as noradrenaline, endothelin-1, and thromboxane and inhibits vascular remodeling through suppressing proliferation and migration of vascular smooth muscle cells and endothelial cells [24–28]. Besides, dietary quercetin supplementation significantly reduces blood glucose level in obese mice and improved hyperinsulinemia in obese rats [29, 30]. In particular, the inhibitory effect of quercetin on intimal hyperplasia has been demonstrated in a rat artery balloon injury model [31–33]. Nevertheless, the pharmacological actions of quercetin on PAH and pulmonary vascular remodeling are unclear. In the present study, we aimed to investigate the effects and mechanisms of quercetin on TGF-*β*1-induced transdifferentiation of human PAECs to smooth muscle-like cells.

## 2. Materials and Methods

### 2.1. Cell Proliferation Assay

PAECs were purchased from the American Type Culture Collection (ATCC, Manassas, VA, USA) and cultured in RPMI1640 medium (Gibco, Life Technologies, Rockville, MD, USA) according to the supplier instructions. The medium was supplemented with 10% fetal bovine serum and 1% penicillin/streptomycin, and cells were used at third passage for experiments.

The CCK-8 assay was used to evaluate PAEC proliferation using a CCK-8 cell proliferation assay kit (Beyotime, Shanghai, China) [34]. PAECs seeded in 96-well plates (10^4^ cells/well) were pretreated with indicated concentrations of quercetin for 1 h and then stimulated with 100 ng/ml TGF-*β*1 for 48 h. Next, 10 *μ*l CCK-8 solution was added to each well and incubated for 4 h. After the medium was removed, 100 *μ*l dimethyl sulfoxide (DMSO) was added to each well. The culture plate was oscillated for 10 min in the shaking table to adequately dissolve the crystals. Finally, cell viability and proliferation were measured by reading the absorbance at 450 nm with Epoch Microplate Spectrophotometer (BioTek, Winooski, VT, USA).

### 2.2. PAEC Identification and Transdifferentiation

PAECs were grown on slides and then incubated with primary antibodies against *α*-SMA (1 : 100, Santa Cruz Biotechnology) and FVIII (1 : 100, Santa Cruz Biotechnology) and then conjugated with FITC- or PE-secondary antibodies for 2 h to specifically identify smooth muscle-like cells and endothelial cells, respectively. 4′,6-Diamidino-2-phenylindole (DAPI) was used to stain nuclei and cells were then observed under a laser scanning confocal microscope (TCS SP5 II, Leica, Wetzlar, Germany) with a 60x oil objective lens to detect the subcellular distribution of target proteins [35]. The fluorescent signals of the corresponding target proteins were collected through the filters with excitation wavelengths of 488 nm (FITC), 488 nm (DAPI), and 543 nm (PE).

### 2.3. BrdU Immunocytochemistry

PAECs grown on 13 mm round coverslips were incubated with a final concentration of 10 *μ*mol/L bromodeoxyuridine (BrdU) (Sigma-Aldrich, St. Louis, MO, USA) for 24 h. The cell cultures were fixed with 4% paraformaldehyde (PFA) in phosphate buffered saline (PBS) (pH 7.6), washed with glycine and PBS respectively, and then immersed in 0.5% Triton-X100 to permeablize the membranes. To denature the DNA strands, the coverslips were incubated with HCl at 0°C, 25°C, and 37°C in sequence. After the cells were treated with sodium-borate buffer (pH 8.4) for 12 min, they were blocked with 2% BSA in PBS for 1 h at room temperature and incubated with anti-BrdU antibodies (1 : 1000, Cell Signaling Technology) overnight at 4°C. Alexa Fluor 488-conjugated anti-mouse IgG (Cell Signaling Technology) was used as the secondary antibody. Finally, the coverslips were stained with DAPI and observed under the laser scanning confocal microscope (Leica, TCS SP5 II, Germany). The signals of BrdU and DAPI were counted in randomly selected four fields, and consequently the ratio of BrdU/DAPI was calculated for each culture in a blinded manner.

### 2.4. Western Blot

Equal amounts of proteins extracted from different groups of PAECs were separated by sodium dodecyl sulfate polyacrylamide gel electrophoresis (SDS-PAGE) and then were transferred to polyvinylidene difluoride membranes (EMD Millipore, Darmstadt, Germany). After blocking with 5% skimmed milk, the membranes were incubated with anti-*α*-SMA antibody (1 : 1000, Santa Cruz Biotechnology), ERK antibody (1 : 1000, Cell Signaling Technology), p-ERK antibody (Thr202/Tyr204, 1 : 2000, Cell Signaling Technology), Akt antibody (1 : 1000, Cell Signaling Technology), p-Akt antibody (Ser473, 1 : 2000, Cell Signaling Technology), GAPDH (1 : 1000, Cell Signaling Technology), and *β*-actin (1 : 1000, Abcam) overnight at 4°C. After washing three times, the membranes were incubated with horseradish peroxidase (HRP) conjugated secondary antibody (1 : 2000, Santa Cruz Biotechnology) and finally detected by enhanced chemiluminescence and analyzed using Kodak 1D 3.5 imaging software (Eastman Kodak, Rochester, NY, USA).

### 2.5. Statistical Analysis

Measurement data were expressed as means ± standard deviation (SD). One-way analysis of variance (ANOVA) was used for comparisons between multiple groups, and the variance homogeneity test was used for variance homogeneity of multiple sample means. In the case of variance homogeneity, the least significant difference (LSD) test was used for comparisons among mean variances of multiple samples. As for variance nonhomogeneity, the Tamhane test was used for comparisons between mean variances of multiple samples and Student's *t*-test was used for intergroup comparison. In addition, SAS 8.0 statistical software was used for statistical processing. A significant difference was observed when *P* < 0.05. QUANTITY ONE software was used to analyze bands in the image to obtain gray value, and the ratio of target protein to internal reference *β*-actin was used for semiquantitative analysis.

## 3. Results

### 3.1. Effect of Quercetin on PAEC Viability and Proliferation

**Figure 1:**
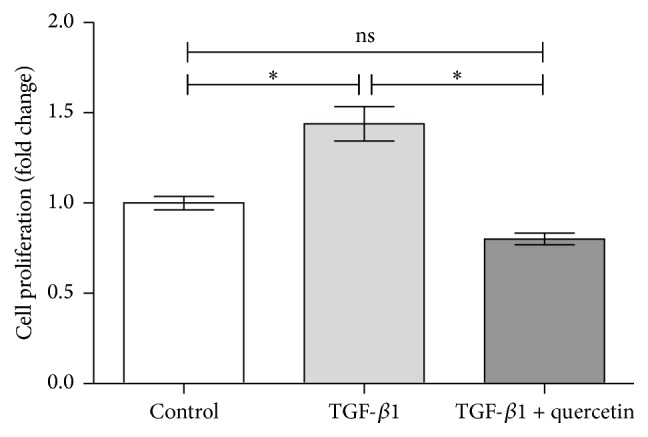
Effect of quercetin on PAEC viability was evaluated by CCK-8 assay (*n* = 4 per group). ^*∗*^*P* < 0.05, the control group versus the TGF-*β*1-induced group and the TGF-*β*1-induced group versus the TGF-*β*1 + quercetin-treated group.

**Figure 2:**
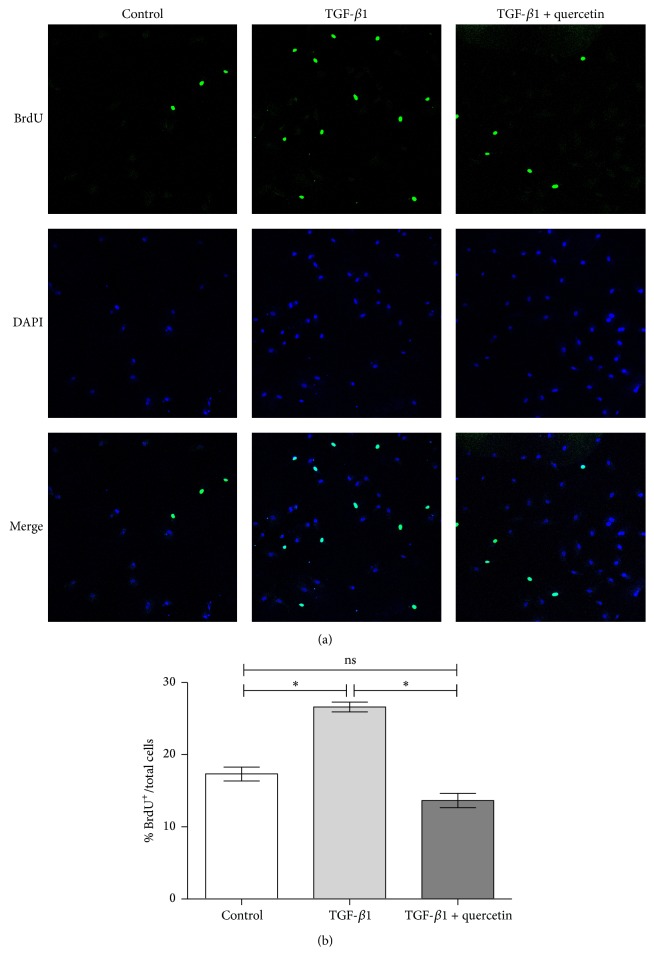
Effect of quercetin on PAEC proliferation by BrdU methods. (a) BrdU immunocytochemistry (scale bar = 100 *μ*m); (b) cell count with BrdU staining, ^*∗*^*P* < 0.05, the control group versus the TGF-*β*1-induced group and the TGF-*β*1-induced group versus the TGF-*β*1 + quercetin-treated group.

To determine the effect of quercetin on TGF-*β*1-induced viability and proliferation of human PAEC, a CCK-8 assay was performed quantificationally. When treated for 48 h, TGF-*β*1 enhanced PAEC proliferation to 1.439-fold (*P* < 0.05), and this viability rate was decreased significantly following quercetin treatment, with distinct differences observed between treatments (Figure 1). To confirm it, the direct outcome of quercetin on cellular proliferation was examined with BrdU incorporation immunocytochemistry (Figures 2(a) and 2(b)). Taken together, the results suggested that quercetin may be a potential antagonist of PAEC excessive growth.

### 3.2. Effect of Quercetin on PAEC Transdifferentiation by Immunofluorescence and Western Blot

**Figure 3:**
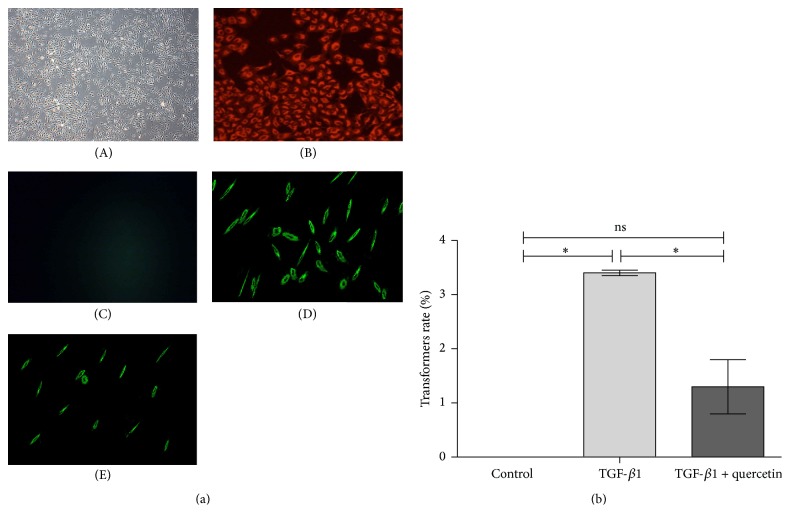
Transdifferentiation of endothelial cells in the cells expressing *α*-SMA induced by TGF-*β*1, as detected by cell immunofluorescence. (a)(A) Blank control PAECs (4x); (a)(B) identification of human PAECs by immunofluorescence. Red staining represents FVIII (10x); (a)(C) blank control PAECs (10x); (a)(D) TGF-*β*1-induced PAECs. Green staining represents *α*-SMA (10x); (a)(E) TGF-*β*1 + quercetin-treated PAECs (10x); (b) conversion rate of PAECs in different groups (*n* = 4 per group), ^*∗*^*P* < 0.05, the control group versus the TGF-*β*1-induced group and the TGF-*β*1-induced group versus the TGF-*β*1 + quercetin-treated group.

As observed using fluorescence microscopy, PAECs stained red for the FITC-labeled FVIII antibody (Figures 3(a)(A) and 3(a)(B)), and green PE-labeled *α*-SMA was absent in these cells (Figure 3(a)(C)). This result confirmed that the population of PAECs was pure. However, after being induced by TGF-*β*1, green-stained cells increased significantly (*P* < 0.05), which became fusiform or polygonal, suggesting these PAECs had transformed into smooth muscle-like cells (Figures 3(a)(D) and 3(b)). Furthermore, when the transdifferentiated cells were treated by quercetin and TGF-*β*1, the number of smooth muscle-like cells in the PAEC culture decreased significantly (*P* < 0.05) (Figure 3(b)), which demonstrated that quercetin could successfully inhibit transdifferentiation of PAECs to smooth muscle-like cells.

**Figure 4:**
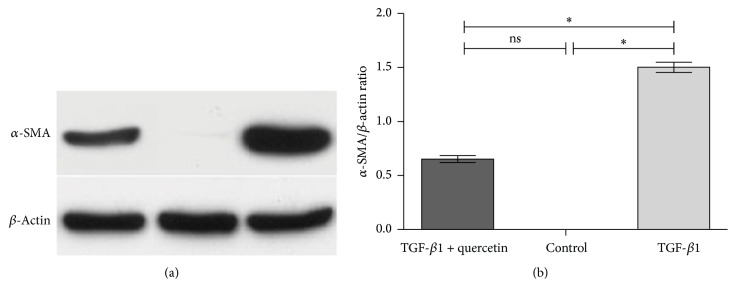
*α*-SMA protein expression in transdifferentiation of endothelial cells. (a) Western blot assay; (b) comparison of gray values of western blot (*n* = 4 per group), ^*∗*^*P* < 0.05, the control group versus the TGF-*β*1-induced group and the TGF-*β*1-induced group versus the TGF-*β*1 + quercetin-treated group.

In PAECs from different treatment groups, *α*-SMA expression was detected by western blot analysis to determine the yield of smooth muscle-like cells (Figure 4(a)). Compared with the blank control group, TGF-*β*1 significantly enhanced *α*-SMA protein (*P* < 0.05) and promoted PAECs to transform into smooth muscle-like cells. Meanwhile, after further treatment with quercetin and TGF-*β*1, *α*-SMA expression was reduced significantly compared with TGF-*β*1 treatment (*P* < 0.05) but was higher than that in the blank group (Figure 4(b)). These data were consistent with the aforementioned immunofluorescence findings, whereby PAECs were induced to transdifferentiate into smooth muscle-like cells, and this cellular process was effectively inhibited by quercetin.

### 3.3. Effect of Quercetin on Akt and Erk1/2 Signal Pathways

**Figure 5:**
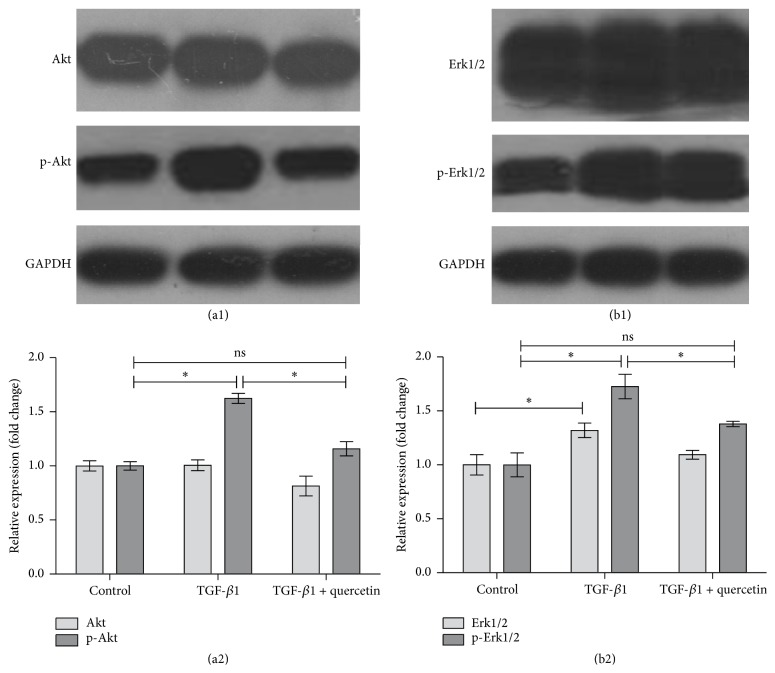
Expression and phosphorylation levels of Akt and Erk1/2 in PAECs in different treatment groups. (a1) Western blot assay of Akt protein; (a2) comparison of gray values of western blot assay of Akt protein (*n* = 4 per group), ^*∗*^*P* < 0.05, the control group versus the TGF-*β*1-induced group and the TGF-*β*1-induced group versus the TGF-*β*1 + quercetin-treated group; (b1) western blot assay of Erk1/2 protein; (b2) comparison of gray values of western blot assay of Erk1/2 protein (*n* = 4 per group), ^*∗*^*P* < 0.05, the control group versus the TGF-*β*1-induced group and the TGF-*β*1-induced group versus the TGF-*β*1 + quercetin-treated group.

To investigate the molecular mechanism involved in how quercetin inhibited TGF-*β*1-induced cell growth, the expression and phosphorylation levels of Akt and Erk1/2 after TGF-*β*1 and quercetin and TGF-*β*1 were determined by western blot (Figure 5). The results showed that TGF-*β*1 promoted Erk1/2 expression rather than Akt, and meanwhile Erk1/2 signaling pathway was inhibited after quercetin treatment. Akt was phosphorylated markedly when PAECs were treated using TGF-*β*1, but it was inhibited dramatically by quercetin approaching the level of control group. In a similar way, Erk1/2 phosphorylation of the cells was enhanced by TGF-*β*1 and then was attenuated significantly by quercetin.

## 4. Discussion

The production of endogenous TGF-*β*1 is promoted during the early stage and implicates the pathogenesis of PAH [36]. It has been revealed that TGF-*β*1 regulates the differentiation and transformation of endothelial cells under some conditions [20]. In this study, we hypothesized that PAEC transdifferentiation is related to TGF-*β*1. Consequently, we found that TGF-*β*1 in vitro triggered and promoted transdifferentiation of PAECs to smooth muscle-like cells and that the new smooth muscle cells causing pulmonary arteriole muscularization could originate from PAECs.

Quercetin can alleviate vascular vasoconstriction [26, 37] and inhibit proliferation and migration of smooth muscle cells and endothelial cells [38–41]. Our results showed that quercetin suppressed TGF-*β*1-induced proliferation and transdifferentiation of PAECs. Comparing with sildenafil, a known inhibitor of hypoxia-induced transdifferentiation of PAECs into smooth muscle-like cells [42], quercetin promised well as a more inexpensive and effective candidate. Thus, the pharmacological action of this natural compound should be further investigated to use as a useful drug.

Meanwhile, we sought to show in Figure 3 the dynamic change of the transition period of endothelial cells into smooth muscle-like cells, at least indicating that some cells have double positive staining for both endothelial/SMCs and the percentage of endothelial-like cells transdifferentiating into smooth muscle-like cells in vitro, but it failed. We are in a puzzle about the cause. However, we think it did not affect our results about transdifferentiation of PAECs into smooth muscle-like cells, and finally the percentage of transformation was obtained by the immunohistochemical analysis.

It would be interesting to know the molecular mechanisms of transdifferentiation and proliferation of PAECs, especially the change and function of signal pathways related to TGF-*β*1. Based on the recent reports about TGF-*β*1-induced cellular proliferation, differentiation, and epithelial-mesenchymal transition (EMT), Akt and ERK1/2 pathways were important downstream modulators activated by TGF-*β*1 [14, 43–45]. In this study, Akt made more positive response to TGF-*β*1 stimulation than that of Ekr1/2, suggesting that Akt may play the crucial role in the PAEC proliferation. Actually Akt has been recognized to be a potent regulator in cell differentiation and EMT processes [46, 47]. When PAECs were treated by quercetin, Akt and Erk1/2 expression reduced with an acute trend although it did not reach the significant level, which may be attributed to the treatment dosage of this drug. However, Akt and Erk1/2 both were phosphorylated markedly when PAECs were treated using TGF-*β*1 and then inhibited dramatically by quercetin, and thus it is reasonable to speculate that phosphorylation activation of Erk/Akt cascades was closely associated with the inhibitory effect of quercetin on TGF-*β*1-induced cell development. These pathways had been demonstrated to mediate the cellular differentiation, proliferation, and survival in many types of cells [48], and here we furthermore reveled that their activation induced by TGF-*β*1 was attenuated under the pretreatment of quercetin in phosphorylation manner.

## 5. Conclusion

Quercetin effectively inhibited TGF-*β*1-induced PAECs proliferation and transdifferentiation into smooth muscle-like cells and downregulated the expression of *α*-SMA protein and activation of Akt/Erk1/2 cascade in the TGF-*β*1-induced PAECs. Therefore, quercetin may be used as a potential drug treating vascular-remodeling related PAH by inhibiting endothelial transdifferentiation possibly via modulating the expression and phosphorylation levels of Akt and Erk1/2 pathways.
